# Trade-Mark Pharmacy

**Published:** 1882-02

**Authors:** Horatio R. Bigelow

**Affiliations:** Washington, D. C.


### ARTICLE III.

**TRADE MARK PHARMACY.** By HORATIO R. BIGELOW, M.D., Washington, D. C.

An attempt is being made to foist upon the American Medical Association the responsibility of discriminating between manufacturing pharmacists, in their endorsement of certain time-honored preparations. Coupled with this is a malevolent and ulterior motive of creating an open rupture between the physician and druggist. The gravity of the two purposes is my excuse for dwelling so frequently upon their ultimate issues, and for publishing my views in the different medical journals of the North, South, East and West. It is of superlative importance that the intelligent practitioner should thoroughly digest these matters in all their manifold phases, especially in those salient features which immediately concern his conceded prerogatives before the next meeting of the Association at St. Paul. As an educated body of men, whose lives are devoted to the amelioration of suffering and whose practice is governed by well-defined laws of self-respect, of courtesy and of justice, we are unwilling to be the puppets of any misguided manager who seeks to press us into his service as actors upon the stage of trade rivalry and trade jealousy. Our calling is professional rather than commercial. If we err in matters touching upon practice, we are to be judged by a tribunal of our peers, and not by laymen, who may be actuated by selfish or ignorant motives. Neither is it consonant with good sense or good taste that those outside the pale of legitimate medicine should dictate to us in the purely professional regulation of fees or of therapeutical polity. As a common brotherhood we are banded together for self-protection, for self-enlightenment, and for the regulation of certain ethical laws which shall govern our inter-communion. We pretend to be able to adapt all of these to suit our necessities, and we are perfectly able to manage our own affairs, without the presumptuous interference of mischief-makers. We are accustomed to construct our prescriptions upon well-thought-out principles, and to order such drugs as experience has taught us are of the greatest efficacy. We recognize our indebtedness to modern pharmacy for valuable additions to our armamentarium, and for the pleasant and palatable preparations of nauseating medicaments. Certain members of our own profession, together with others honored in society, recognized as men of high sense of honor, and many of them graduates of our learned universities, have entered into business for the sole purpose of furnishing pure drugs of an unalterable standard, in such forms as may not disgust our patients. These elegant products of their zeal and industry have added luster to our practice, and have proved of measureless value. The good name, fame and personal reputation of the manufacturer is indissolubly connected with his wares. The convalescence of our patients depends upon the strict purity and unvarying quantity of these preparations: our own reputation hinges upon our faith in them. To protect all these common interests, and to prevent fraud and deceit, the pharmacist trade-marks the products of his laboratory. What gross injustice and what danger to us, to our patients, and to the manufacturer, would result upon a discontinuance of the system. The law is protective in all its ramifications, just as the sworn policy of the Government is protective. The Government is a mere verbal synonym of an aggregation of individuals. The protective tariff is framed in the interest of home industries. So the individual member of this corporate body protects his industry, and the law and the people endorse his action. Physicians must recognize that for them there can be no other protection than this trade-mark system. It is the only safe guarantee. Can it be supposed that the nestors of medicine—the names that we most love to honor—would endorse in medical literature, these additions to elegant pharmacy, simply for any selfish delight in seeing their names in print? It would be well for us and for our children, if this incoming wave of grotesque "New Remedies" could be stayed and if the few deserving new discoveries, could come to us guaranteed by trade-mark as to purity and by endorsement as to efficacy. Let us take personal interest in the matter, and protect, as we are in duty bounden, the common interest of the profession.

